# Challenges in Managing Cephalic Arch Stenosis: A Case of Stent Fracture and Lessons Learned

**DOI:** 10.7759/cureus.77290

**Published:** 2025-01-11

**Authors:** Susumu Doita, Yosuke Takahashi, Eisuke Nakamura, Kazufumi Sakurama

**Affiliations:** 1 Department of Gastroenterological Surgery, Okayama University Graduate School of Medicine, Dentistry, and Pharmaceutical Sciences, Okayama, JPN; 2 Department of Surgery, Shigei Medical Research Hospital, Okayama, JPN

**Keywords:** balloon-expandable stent, cephalic arch stenosis, hemodialysis, shoulder movements, stent fracture

## Abstract

Cephalic arch stenosis (CAS) is a common cause of vascular access dysfunction in patients undergoing hemodialysis. This report describes a case of recurrent stent fractures and restenosis following endovascular treatment for CAS. A 34-year-old man with diabetic nephropathy and chronic hemodialysis underwent multiple stent placements for CAS. Complications, including stent entanglement during balloon angioplasty and stent fracture due to the cephalic arch’s anatomical characteristics, necessitated surgical intervention. This case highlights two critical considerations: the unsuitability of balloon-expandable stents for CAS, as they lack resilience to deformation caused by shoulder movements, and the importance of selecting appropriate balloons to minimize stent damage. The unique anatomical characteristics of the cephalic arch increase the risk of stent bending and fracture, emphasizing the need for careful device selection. This case increases the awareness of stent placement for CAS.

## Introduction

Hemodialysis is the most common treatment for patients with end-stage renal disease. Despite being the ideal route, arteriovenous hemodialysis access is still plagued by frequent dysfunction, including thrombosis and stenosis. The cephalic arch is a common site of vascular access dysfunction. The pathophysiology of cephalic arch stenosis (CAS) remains poorly understood. Several mechanisms have been proposed, including altered blood flow, extrinsic compression of the vessel by fascia and pectoralis major muscle, the anatomy of the cephalic arch and the entry angle of the cephalic vein as it enters the axillary vein, and turbulent and presence of multiple valves [1]. Hence, we need to take these characteristics into consideration when treating CAS.

The current management for CAS includes transluminal balloon angioplasty, stent grafts, bare metal stents, cutting balloon angioplasty, endovascular banding, and surgical procedures [2]. Appropriate management for CAS is highly debatable and depends on the approach of patients and interventionists. In the context of stent placement for CAS, the optimal stent type remains uncertain. As stent-related treatments have become increasingly utilized, it is crucial to discuss their associated complications, efficacy, and long-term outcomes. Here, we report a case of stent fracture in the treatment of CAS.

## Case presentation

**Figure 1:**
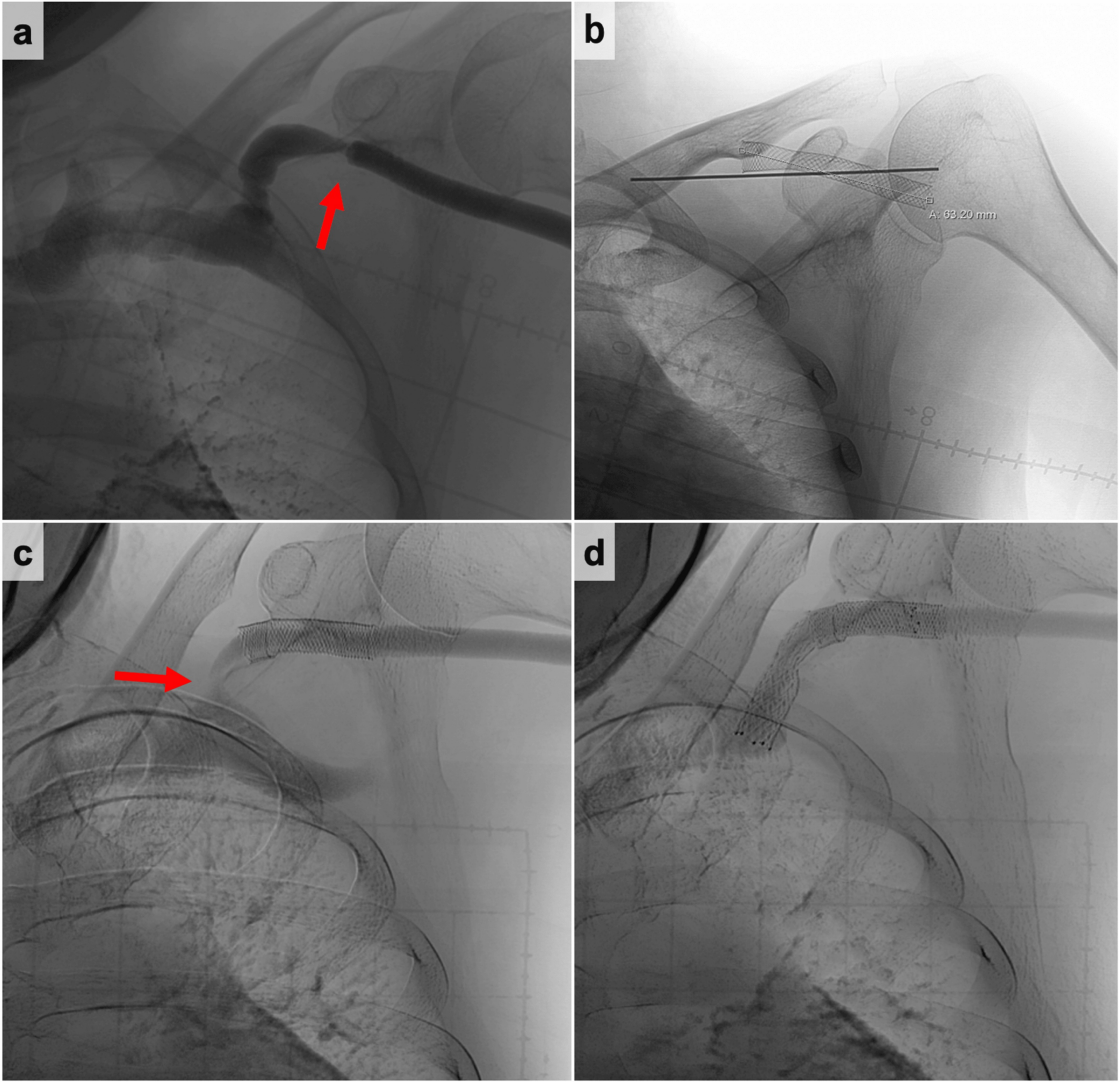
The digital subtraction angiography (DSA) findings. (a) Initial DSA showing cephalic vein stenosis (red arrow); (b)first stent placement addressing cephalic vein stenosis; (c) follow-up DSA showing recurrent cephalic arch stenosis (red arrow); and (d) second stent placement for recurrent cephalic vein stenosis. DSA, digital subtraction angiography

**Figure 2:**
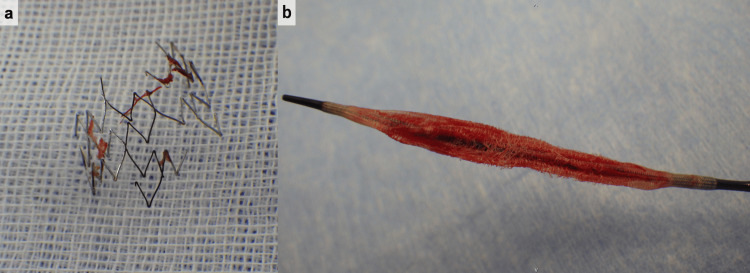
Pictures of the damaged stent and balloon. (a) The stent is damaged and partially retrieved. (b) The damaged balloon with a suture wrap.

**Figure 3:**
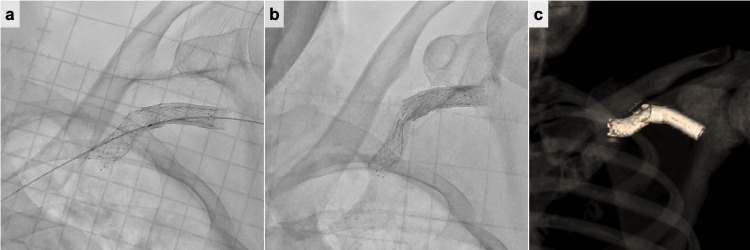
The digital subtraction angiography (DSA) and CT findings. (a) The balloon-expandable covered stent was placed to overlap the damaged stent. (b) Follow-up DSA showed recurrent stent stenosis. (c) 3D-CT revealed the stent fracture. DSA, digital subtraction angiography; CT, computed tomography

A 34-year-old man receiving chronic hemodialysis due to diabetic nephropathy was referred to our hospital for hemodialysis vascular access dysfunction. Hemodialysis was started eight years ago. The arteriovenous fistula was created in the left forearm. The digital subtraction angiography (DSA) showed cephalic vein stenosis (Figure 1a). The stenosis was adequately expanded using a 6-mm balloon (YOROI; Kaneka Corp, Osaka, Japan), but angiography revealed immediate restenosis. Therefore, we deployed a 9 × 35 mm self-expanding stent (Wallstent; Boston Scientific, Natick, MA) (Figure 1b). Seven years after the stent placement, ultrasonography showed 1.4 × 3.0 mm CAS. The DSA revealed stenosis on the central side of the previous stent placement (Figure 1c). Therefore, the self-expanding stent (Epic™ stent; Boston Scientific, Natick, MA) was placed in continuity with the previous stent (Figure 1d). Two years after the second stent placement, acute hemodialysis access thrombosis was found. A stiff 0.018 wire (FUGA™; Boston Scientific, Natick, MA, USA) was gently advanced across the lesion, and the severe stenosis in the peripheral vein was adequately expanded by using a 7-mm balloon (YOROI; Kaneka Corp, Osaka, Japan). The angiography, after expanding, showed the in-stent stenosis. A 7-mm balloon (YOROI; Kaneka Corp, Osaka, Japan) expanded in-stent stenosis. However, the outer thread of the expanded balloon entangled with the stent, and as it was withdrawn, part of the stent was damaged, and the thread around the balloon was partially torn (Figure 2). Because there was the possibility of the stent dislodgement due to the stent fracture, the balloon-expandable covered stent (Gore Viabahn VBX 10× 59 mm; W. L. Gore & Associates, DE) was placed to overlap the damaged stent (Figure 3a). One year later, the stent restenosis was found (Figure 3b), and we expanded it by using an 8-mm balloon (Conquest; C. R. Bard, Osaka, Japan). The vascular access dysfunction was improved, but two weeks later, an increase in intravascular pressure during dialysis was noted. Computed tomography (CT) was conducted and revealed the stent stenosis again (Figure 3c). Due to recurrent stent stenosis in a short period, surgical removal was deemed preferable. Under local anesthesia, the proximal cephalic vein was exposed and transected proximally. We attempted to remove the stent through the transected end, but complete removal was not achieved. We decided to conduct the cephalic vein transposition (CVT) procedure. The proximal cephalic vein was mobilized to ensure adequate length for transposition to the axilla. Another incision was made to expose the proximal axillary vein. The cephalic vein stamp was closed, and the proximal cephalic vein was subcutaneously transposed. The cephalic vein was anastomosed to the axillary vein in an end-to-side anastomosis. After the surgical treatment, intravascular pressure was improved. The patient was doing well at the outpatient follow-up at two years, with no vascular access dysfunction.

## Discussion

This case highlights two important clinical considerations: (1) balloon-expandable stent grafts should not be chosen for the treatment of CAS and (2) the choice of the balloon for stent expansion is important.

First, balloon-expandable stent grafts should not be chosen for the treatment of CAS. The cephalic arch refers to the terminal segment of the cephalic vein before draining into the initial portion of the axillary vein. The cephalic vein courses along the lateral aspect of the biceps, coursing toward the pectoralis major muscle as it enters the deltopectoral groove (a triangular space formed by the adjacent borders of the deltoid and pectoralis major muscles). It then passes under the clavicle, makes a sharp turn to pierce the clavipectoral fascia, and terminates by draining into the axillary vein [1]. The cephalic arch is surrounded by various layers of tissue, making it susceptible to compression and torsion from shoulder movement. A balloon-expandable stent does not naturally return to its original shape once deformed by applied force. If placed in a cephalic arch, there is a high risk of permanent damage from shoulder movement. Additionally, some researchers reported that self-expanding stents were preferred because of their low migration risk [3]. Hence, we do not recommend using balloon-expandable stents for the treatment of CAS.

Second, the choice of balloon for stent expansion is important. The unique anatomical characteristics of the cephalic arch present challenges for stent placement, as the stent placed in CAS is prone to bending [4,5]. When the bare metal stent is bent even slightly, the stent will protrude into the vessel lumen and become trapped easily. The balloon with a suture wrap such as the YOROI balloon has the risk of stent entanglement and damage. In contrast, Conquest™ 40 PTA dilatation catheter, recommended for post-dilation of stents and stents graft, is made of composite material with a fiber design, which makes them less susceptible to rupture and entanglement by calcified lesions or stent struts. This case proposes the importance of careful device selection, particularly in anatomically complex areas such as the cephalic arch, to prevent stent deformation and associated complications.

Angioplasty and stent placement for CAS have already been reported. Dukkipati et al. reported that stent placement prolongs the patency compared to angioplasty alone [3,6]. Huang et al. represented that undersized stent grafts in patients with CAS showed significantly higher primary stent and access patency rates [5]. Mehta et al. conducted a study to compare the double mesh nitinol stent versus the self-expanding stent-graft (SES) in CAS and represented that SES showed statistically significant higher primary patency [4]. The appropriate management option is highly debatable. In this case, recurrent stent fractures were associated with symptoms such as vascular access dysfunction and increased intravascular pressure during dialysis sessions. Imaging studies, including venography and ultrasound, confirmed restenosis and stent fractures, highlighting the importance of routine surveillance in similar cases.

## Conclusions

Self-expanding stents are preferable to balloon expandable stents for the treatment of CAS due to their flexibility and durability, which may help mitigate the risk of stent fracture caused by the unique anatomical characteristics of the cephalic arch. Careful consideration of the stent type and appropriate balloon selection is crucial for successful outcomes. While angioplasty and stent placement remain valuable treatment options, further research is warranted to investigate the long-term outcomes of different stent types and techniques, including optimal stent design and deployment strategies, to further refine the management of CAS.
